# Childhood Affective Indicators of Risk for Adulthood Psychopathology: The New York High-Risk Project Findings

**DOI:** 10.20900/jpbs.20180004

**Published:** 2018-06-05

**Authors:** Diane C. Gooding, Carolyn Zahn-Waxler, Sharee N. Light, Clarice J. Kestenbaum, L. Erlenmeyer-Kimling

**Affiliations:** 1 Department of Psychology, University of Wisconsin-Madison, Madison, WI 53706, USA; 2 Department of Psychiatry, University of Wisconsin-Madison, Madison, WI 53706, USA; 3 Center for Healthy Minds, University of Wisconsin-Madison, Madison, WI 53703, USA; 4 Department of Psychology, Georgia State University, Atlanta, GA 30302, USA; 5 College of Physicians and Surgeons, Columbia University, New York, NY 10032, USA; 6 Department of Child Psychiatry, New York State Psychiatric Institute, New York, NY, 10032, USA; 7 Department of Psychiatric and Medical Genetics, New York State Psychiatric Institute, New York, NY, 10032, USA.

**Keywords:** genetic high-risk, schizophrenia, affect, emotional expressivity

## Abstract

There are relatively few investigations of the emotion expressivity of children at risk for the later development of schizophrenia and schizophrenia-spectrum disorders. Using data from the New York High-Risk Project, we compared children’s emotional expressivity during a semi-structured videotaped interview. Data were coded for 173 child subjects: 61 with schizophrenic parents (HRSz); 54 with affectively ill parents (HRAff); and 58 with psychiatrically “normal” parents (NC). A child’s affective responses were rated for the presence of discrete positive, negative, or neutral emotions by coders naive to group membership. Responses were also rated for anxiety, flat affect, inappropriate affect, and emotional withdrawal/disengagement. Compared with the two other two groups, HRSz children displayed significantly more negative affect in response to questions regarding their most negative experiences and, when questioned about their self-concept, they displayed significantly less positive affect. Both HRSz and HRAff children showed more inappropriate affect than NC children. Significantly more HRSz children were rated as demonstrating a lack of emotional engagement. Children making inappropriate displays of positive affect while discussing a negative topic were most likely to manifest a psychiatric disorder as an adult. These findings suggest that inappropriate affect may be a nonspecific indicator of risk for psychopathology. Emotional withdrawal in childhood may be a potential indicator of risk for schizophrenia.

## 1 INTRODUCTION

There is longstanding interest in children at risk for schizophrenia. This is largely due to recognition that the years prior to schizophrenic psychoses manifestation in adulthood are characterized by atypical and/or abnormal development ^[1]^. The notion that adulthood psychopathology is preceded by signs of childhood behavioral and/or emotional disturbance is consistent with conceptualizations of schizophrenia-related psychoses as neurodevelopmental in origin ^[2]^. Since Kraepelin ^[3]^, affective disturbances have been considered central to schizophrenia. Affective disturbances in schizophrenia include affective experience, and expression, impairments such as anhedonia and flat, or blunted, affect, increased emotional arousal and reactivity, and anxiety symptoms ^[4]^. As emotional disturbances and affective dysregulation are observed in various forms of psychopathology, it is useful to examine whether specific aspects of emotional dysfunction associate with heightened risk for schizophrenia.

There are relatively few investigations into the emotional expressivity of children at risk for the later development of schizophrenia and schizophrenia-spectrum disorders. Using childhood home movies of schizophrenia patients and their healthy siblings, Walker, and her colleagues ^[5,6]^, compared the facial expressions of emotions from infancy through adolescence. This archival-observational method proved quite fruitful in that it revealed that both male and female pre-schizophrenic children showed greater negative emotion than same-gender siblings who did not later develop schizophrenia. Comparative analyses also revealed that pre-schizophrenic girls, but not boys, displayed a significantly lower proportional duration of joy responses than their healthy siblings.

Videotaped data from the Copenhagen High-Risk Study ^[7]^ revealed that individuals who developed schizophrenia in adulthood could be discriminated from those who did not on the basis of their lunchtime eating behavior. Specifically, children who later developed schizophrenia smiled and vocalized less, whether initiated by themselves or another child, than children who either developed a non-schizophrenic form of psychopathology, or did not develop a mental illness.

There are also relevant prior findings from the New York High-Risk Project (NYHRP). The NYHRP is a prospective, longitudinal, high-risk study of initially unaffected and untreated offspring of individuals with schizophrenia, major affective disorders, and those without psychiatric disorders. The project consisted of two independent samples—Sample A and Sample B—who were followed continuously since recruitment through the period of greatest lifetime risk for schizophrenia, up through a mean age of 42 years. Based upon a subset of Sample B, Dworkin *et al.* ^[8]^ found that ratings of the videotaped interviews of the high-risk offspring revealed significant differences in social competence, such as emotional withdrawal, as defined by the Premorbid Adjustment Scale ^[9]^; at ages 12 and 15, and affective flattening at 15. In another analysis of Sample B ^[10]^, the children of schizophrenia patients showed significantly fewer smiles per minute during adolescence and greater affective flattening. It is noteworthy that the reports by Dworkin and colleagues were based on a subset of the NYHRP dataset, and before all the children had passed through the period of greatest risk.

The present study is an extension of the earlier analyses using the NYHRP interview data. In addition to comparing the offspring of parents with psychiatric disorders in terms of their emotional expressions in response to questions during a semi-structured interview, we also compared offspring groups in terms of the affective quality of the child/interviewer interaction. The degree of child emotional engagement versus emotionally withdrawal was examined. The present report extends earlier work by following the subjects through the period of greatest lifetime risk for schizophrenia.

We were interested in exploring whether an association between increased negative displays and risk for the later development of schizophrenia would be found. Adult-onset schizophrenia patients display fewer negative facial displays compared to healthy controls despite experiencing the same amount of emotion ^[11]^. When comparing young offspring of schizophrenic, depressed, and well mothers, Goodman ^[12]^ observed less negative affect among the offspring of mothers with schizophrenia. Walker *et al.* observed that pre-schizophrenic children exhibited more negative emotional facial expressions than their unaffected siblings. We hypothesized that children of schizophrenia patients would display more negative affect than the other children based upon prior NYHRP findings based on a subset of the sample and findings based of the Walker’s archival-observational study.

We hypothesized that we would find an association between reduced positive displays and the risk for the later development of schizophrenia. The basis for this hypothesis is both conceptual and empirical. First, schizophrenia patients display fewer positive facial displays compared with non-schizophrenia patients in response to a range of emotion-eliciting stimuli ^[11]^. Secondly, we reasoned that the offspring of schizophrenia patients might show similar expressive affectivity as their mothers with schizophrenia, due to genetic, social learning, and/or transactional developmental processes. Finally, fewer positive facial displays among those at heightened risk for the later development of schizophrenia would be consistent with the earlier findings of Walker and colleagues.

We also included other affective indicators that could be viewed as interpersonally maladaptive, such as low positive affect, anxiety, inappropriate affect, and/or flat affect. We examined whether any of these affective indicators of risk for the later development of psychopathology would be relatively specific for schizophrenia. As children of mothers with depression are at risk for the later development of psychopathology ^[13,14]^, that offspring group served as a psychiatric comparison.

We hypothesized that both groups of children of psychiatric patients would display higher rates of anxiety relative to children of psychiatrically healthy women. Extant research literature provides additional support for our a priori hypothesis. Birth cohort studies such as ^[15]^ indicate that anxiety is a childhood predictor of later adult psychiatric disorder. Epidemiological studies ^[16]^ identify anxiety symptoms as a risk factor in the development of adult schizophrenia. Children and adolescents at genetic risk for schizophrenia had significantly higher anxiety scores than healthy comparison peers ^[17]^. Children of unipolar depressed women are reported as showing significantly more internalizing behavior problems, including anxiety, than other groups of children such as children of bipolar women ^[18]^ and children of psychiatrically healthy women ^[19,20]^.

We also reasoned that the increased rates of anxiety might partly reflect the additional stress associated with living with a parent suffering from a chronic illness and dealing with functional impairments.

The affective system, thought to be composed of neural processes, expressive behavior, and subjective experience, interacts and is coordinated with the cognitive system and affective-cognitive control structures during typical development ^[21]^. If schizophrenia is a disorder of “pandysmaturation” ^[22]^ or “cognitive dysmetria” ^[23]^, then individuals at heightened genetic risk for the disorder may be expected to display less control over the processes regulating the output of their emotional system. Individuals with less control over such processes, as well as less control over the coordination between their affective and developing cognitive systems, are more likely to manifest disturbances such as faulty connection between their emotional display and the emotions expressed, *i.e.*, inappropriate affect. We hypothesized that individuals with a genetic diathesis for schizophrenia (*i.e.*, the offspring of schizophrenic patients) might be more likely to display inappropriate affect.

Blunted, or flat, affect, a reduction in the intensity of expressed feeling, is considered a core schizophrenia symptom ^[4]^. In the New York Infant Study, one of the earliest indications of an association between emotional abnormalities and a schizophrenia diathesis, four out of five of the offspring of a mother with schizophrenia displayed affect described as flat, detached, or distant ^[24]^.

Earlier investigators ^[25–27]^ reported associations between affective deficits and neuromotor dysfunction. More recently, Lee *et al.* ^[28]^ used functional brain imaging to demonstrate how schizophrenia patients with flat affect showed functional disturbance in the mirror neuron system. We expected that a larger proportion of HRSz offspring would display flat affect compared to the other offspring groups.

We were interested in examining whether high-risk offspring displayed emotional withdrawal. Social withdrawal was previously observed among school-age children—especially males who were at genetic risk for schizophrenia—in both the Jerusalem Infant Development Study and the Israeli High-Risk Study ^[29,30]^. Prior investigations, based on the National Child Development Study using teacher ratings ^[31]^, and a prospective followup study of offspring of mothers with psychotic disorders ^[32]^ suggested that childhood social inhibition and emotional withdrawal might be an antecedent to schizophrenia. Retrospective parental and caregiver reports ^[33,34]^ indicate that emotional and social withdrawal in mid-childhood associates with heightened risk for a adulthood schizophrenia-related outcome. Moreover, prodromal studies ^[35,36]^ indicate that individuals with non-affective psychosis typically display social withdrawal in the years preceding their first psychotic episode. We thought it likely that a greater proportion of the schizophrenia offspring group would display emotional withdrawal compared to the other offspring groups.

## 2 MATERIALS AND METHODS

### 2.1 New York high-risk project

All participants were drawn from the New York High-Risk Project (NYHRP). The NYHRP was a prospective, longitudinal study of the offspring of schizophrenic, affectively ill, and psychiatrically normal parents (HRSz, HRAff, and NC offspring groups, respectively). Recruitment procedures, parental diagnoses, and longitudinal follow-up details appear elsewhere ^[37–39]^. Mentally ill parents were identified through consecutive admissions at several large New York State psychiatric facilities. They were recruited if they met the study criteria, including, but not limited to, having at least one biological child aged 7–12 inclusive. All offspring were Caucasian, English-speaking, and free of mental retardation/intellectual disability, major psychiatric disorders or treatment for emotional problems at the time of recruitment in 1971–1972 (Sample A) or 1977–1979 (Sample B) at ages 9.5 ± 1.7 and 9.0 ± 1.8 (mean ± SD), respectively.

Two independent samples were recruited. The addition of a second sample allowed a test of the replicability of findings from the first sample as well as a way of testing additional hypotheses suggested by data generated from Sample A. Since inception the study has had 7 rounds of assessments, with telephone interviews at least once annually. Offspring were followed from mid-childhood (7–12) through mid-adulthood. After a complete description of the study, written informed consent was obtained from the parents for themselves and their children starting in round 1 and individually from the children who had reached 18 in subsequent rounds. Institutional Review Board approval was obtained for each phase of the NYHRP.

### 2.2 Participants

Our subjects were drawn from both independent samples of the New York High-Risk Project. We had the usable videotaped interviews from both mid-childhood and adolescence and diagnostic interviews from adulthood for 173 offspring. The combined sample consisted of 61 offspring of schizophrenic parents (HRSz group); 54 offspring of affectively ill parents (HRAff group); and 58 offspring of psychiatrically normal parents (NC group). Child mean age at the time of the videotaped interviews was 10.65 years (range, 7–15).

### 2.3 Videotaped childhood interviews

During the first, second, and third testing rounds, child psychiatrists who were naive to subject group membership administered 30 min semi-structured videotaped interviews pertaining to subject family and home life, school and extracurricular activities, fantasy life, and self-concept. These interviews were subsequently rated for psychopathology and adaptive functioning using the Mental Health Assessment Form (MHAF) ^[40]^. These videotapes were later transferred to DVD and coded by trained raters who were naive to group membership and/or adult outcome. A child’s affect was rated during selected segments of the interviews.

### 2.4 Selection of coding segments

The primary author, who was naive to group membership and adulthood outcome diagnoses, was responsible selecting which segments were to be coded. Before actual rating had begun, the project coordinator provided the primary author with a representative sampling of videotaped interviews. These videotapes consisted mostly of target subjects’ siblings (*n* = 20) who were not included in the final sample. The objective was to select questions that would be the most likely to elicit positive, or negative, emotional responses from the child and that were queried consistently and frequently enough to insure that there would be sufficient data to code. We selected certain questions that were expected to elicit positive affect (e.g., “What was the best thing that ever happened to you? What was the happiest thing that ever happened to you?”) or negative affect (e.g., “What was the worst thing that ever happened to you? What’s the saddest thing that ever happened to you?”). We also selected questions that were likely to elicit a positive, negative, or neutral, affective response. These types of questions tended to vary by age. Younger children were asked (“Are you happy or are you sad most of the time” whereas older children were asked (“What’s your general mood like?”). All children were asked “What would you change about yourself?” or “Is there anything that you would change about yourself?” and all children were asked either about day dreaming/mind-wandering or dreaming at night. The number of subjects who were rated on each question varied somewhat as a result of videotape deterioration, as well as differences in quality of the available recording ^[41]^. Only one interview session for each child was used. The session used was the session that had the greatest number of codable segments (*i.e.*, questions). It is noteworthy that mental health professionals administering the MHAF were querying the children about their experiences, while the research assistants were rating the child’s affective expressions as they were recounting those experiences.

### 2.5 Coding system and training in coding procedures

After careful consideration of extant coding systems for rating affective responses in children based primarily upon micro-analysis of musculature, we combined that information with vocal prosody ratings and overall behavioral observations. Research assistants who rated the offspring’s emotional expressivity received extensive training with Ekman faces, as well as additional consultation with an expert in child emotions. Further training was conducted using videotapes of study participant siblings. Inter-rater reliability was determined by having three coders independently code 50% of these video records. After attaining an average 0.85 agreement, participant video coding proceeded. In order to assess inter-rater reliability, 10% of all interviews were randomly selected and recoded by a second observer. Calibration meetings were held at regular intervals with the primary trainer. Inter-rater reliability for specific emotions and qualitative ratings was assessed by calculating intra-class correlations and kappa statistics, respectively ^[42]^.

### 2.6 Ratings of emotional expressivity

Initially, although individual emotions were identified and coded, they were classified as positive, negative, or neutral ^[43]^. The following emotions were coded: positive (happiness, joy/pleasure, relief, satisfaction/pride, excitement, confidence, surprise, humor, and interest); neutral (calmness); and, negative (tension, worry, hostility/antipathy, boredom/apathy, annoyance/irritation, frustration/ anger, sadness/anguish, pain/distress, and disgust). Behavioral anchors at each point of emotional intensity were provided.

All non-neutral emotional expressions were rated on 3 point scales, which were coded as mild, moderate, and severe expressions, as ±1, ±2, and ±3, respectively. The positive and negative signs denoting emotional valence. For example, moderate affect was rated as “2”; if it was positive, it was a “+2” and if negative, it was a “−2”.

### 2.7 Ratings for the presence of maladaptive affective and social behaviors

A child’s affective, and social, behaviors during selected interview segments were also rated for the presence of possible indicators of later psychopathological risk, such as anxiety, flat affect, inappropriate affect, and emotional withdrawal. If the behavior was rated as “present”, it was also rated in terms of intensity. Anxiety behavioral indices were operationally defined as including excessive fidgeting, lip- and/or nail biting, and playing with the hair or the microphone. Anxiety could therefore be rated as mild, moderate, or severe. We relied upon 3 of the 4 SANS indices ^[44]^ for the operational definition of flat affect, *i.e.*, a child was rated as displaying flat affect when they displayed unchanging facial expression, affective non-responsivity, and/or lack of vocal inflection. A child was determined to demonstrate inappropriate affect if there was a discrepancy between their facial expression and the topic discussed. The most frequent type of inappropriate affect was the display of positive affect while discussing a negatively-toned topic, e.g., smiling while talking about riding one’s bicycle into a concrete wall. Emotional withdrawal was operationalized as behavioral evidence of a lack of emotional engagement during the interviews, including, but not limited to, poor eye contact, or the child appearing distant, inattentive, cold, or aloof. An extreme example of emotional withdrawal would be a total lack of communication. A child’s expressiveness affect ratings during the interview were related to the topic discussed and the range of feelings expressed. Emotional withdrawal ratings were based upon the quality of child-interviewer relatedness during the interview.

Emotional withdrawal was rated on a dichotomous scale, “0” or “1”, for the presence or absence, respectively, of emotional engagement. If a child was observed to display emotional withdrawal, then they were also rated for the degree to which they displayed lack of emotional engagement, ranging from mild to severe.

### 2.8 Assessment of Outcome psychiatric diagnoses

During rounds 4 through 6, the Schedule for Affective Disorders and Schizophrenia Lifetime Version (SADS-L) ^[45]^ was administered to all participants 18, and older, by trained clinical psychologists and psychiatric social workers naive to parental diagnostic group and offspring outcome diagnoses in adulthood in order to assess Axis I disorders based on the Research Diagnostic Criteria ^[46]^. Final diagnostic evaluations were conducted in 2002, at mean ages 39.4 ± 1.8 (Sample A) and 34.1 ± 2.3 (Sample B). Detailed diagnostic evaluation descriptions were given previously ^[37–39]^. In addition to the SADS-L, the diagnostic evaluation included all other clinical data: research interviews, psychiatric hospital records, and when relevant, therapist notes and comments.

Adulthood Axis I disorders were categorized according to the following hierarchy : (a) schizophrenia-related psychoses (SRP; including schizophrenia, unspecified functional psychosis, and schizoaffective disorder, mainly schizophrenia, as defined in the RDC); (b) affective psychosis (psychotic major depression, bipolar I with psychosis, bipolar II with psychosis, manic psychosis, and schizoaffective disorder, mainly affective, as defined in the RDC); (c) non-psychotic affective disorders (e.g., major depression); (d) other major Axis I disorders (e.g., anxiety disorders, substance abuse disorders); (e) drug-related psychosis; and, (f) no disorder. Participants were also evaluated for the presence of schizotypal features using the Personality Disorders Examination (PDE) ^[47]^, a semi-structured clinical interview designed to assess all DSM-III-R Axis II disorders. The diagnostic algorithm provided in the PDE was used ^[48]^.

### 2.9 Data reduction and statistical analysis

**Fig. 1:**
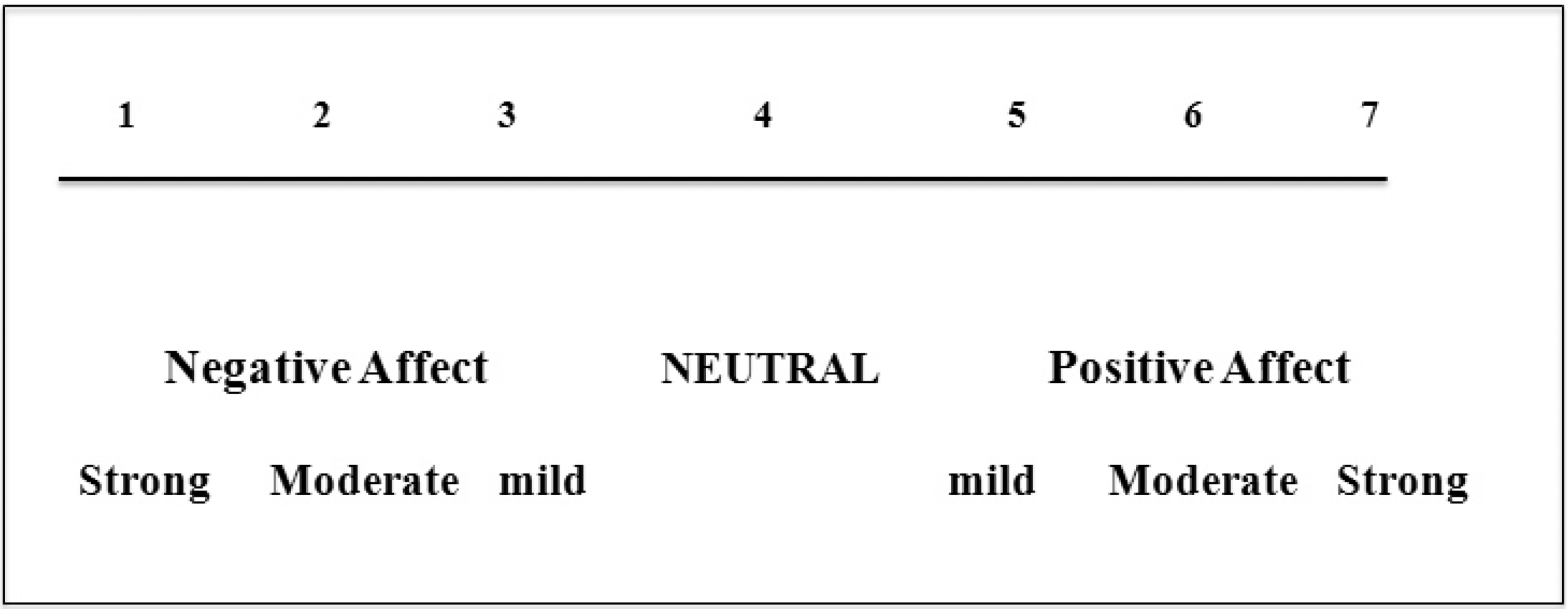
Positive and negative affect were initially rated separately, on a scale of 1 (mild) to 3 (strong) for each, with the positive or negative sign denoting the valence. The coding ratings were subsequently placed on a singular scale to facilitate comparisons across questions. As can be seen, 1 was strongly negative, 4 was neutral, and 7 was strongly positive.

After affective expressivity ratings were made, they were recoded onto a single 7-point rating scale to facilitate cross-question comparisons (Fig. 1). On this 7-point scale, the recoded scores ranged from 1, denoting a strongly negative emotional expression to 7, signifying a strongly positive emotional expression. This method allows for simultaneously conveying valence and intensity of the child’s response to each of the questions posed.

Interrater agreement for the video ratings was calculated using Cohen’s kappa ^[42]^. We used univariate analysis of variance (ANOVA) tests to compare the groups in terms of mean levels of affective expressivity in responses to questions. Tukey’s tests were used for follow-up analyses. In order to compare groups in terms of the presence of anxiety, flat affect, inappropriate affect, and/or emotional withdrawal, we used chi-square analysis. All statistical tests of group differences were two-tailed, with the significance (alpha) levels set at *p* = 0.05.

## 3 RESULTS

### 3.1 Demographic characteristics

Age and gender breakdowns for each offspring group appear in Table 1. Mean age among three groups did not differ, *F* (2, 170) = 0.19, n.s.. The mean age of the sample was 10.65 (±1.8) years. The HRAff group had a higher proportion of females than the other two groups, Χ^2^ (2) = 14.58, *p* < 0. 01 ^[49]^.

### 3.2 Group comparisons by parental risk status

ICC’s for the ratings of positive, neutral, and negative emotional displays were 0.96, 0.97, and 0.80, respectively. Emotion expressivity mean ratings by offspring group and interview question appear in Table 2. Children tended to show more positive emotions in response to questions pertaining to their best, or happiest, experiences. The groups did not differ in positive emotion demonstrations in response to questions regarding their most positive experiences, *F* (2, 93) = 1.96, n.s. All groups exhibited more negative emotions in response to questions pertaining to their worst, or saddest, experience. HRSz children displayed significantly more negative affect than the other children in response to questions regarding their most negative experiences, *F* (2, 136) = 4.35, *p* = 0.02, η^2^ = 0.06. The HRSz group displayed more negative affect than the HRAff group [*t* (82) = −2.10, *p* = 0.04] and the NC group [*t* (93) = −3.05, *p* = 0.003].

We also observed a significant group difference when the children were questioned about their self-concept, *i.e.*, how much did they like themselves or what would they change about themselves. The HRSz group showed significantly less positive affect than the other two groups, *F* (2, 78) = 4.59, *p* = 0.01, η^2^ = 0.11. The HRSz children displayed less positive affect than either the HRAff children [*t* (50) = −2.85, *p* = 0.01] or the NC children [*t* (60) = −2.33, *p* = 0.02].

The three groups did not differ significantly in affective display while describing their general mood, *F* (2, 92) = 0.94, n.s. The three risk groups did not differ in their affective responses to non-emotionally-charged questions (e.g., describing day dreaming or mind-wandering [*F* (2, 66) = 0.43, n.s.]), or when describing dream content [*F* (2, 115) = 0.74, n.s.].

Table 3 provides the number of participants in each offspring group who evidenced anxiety, flat affect, inappropriate affect, and/or emotional withdrawal during the MHAF coded segments. Kappa value for the anxiety ratings was 0.87. Over 75% of the children in each offspring group showed some signs of anxiety while responding to at least one of the coded questions. Overall, the groups did not differ in terms of the proportion of children evidencing anxiety. However, we observed a trend in the two high risk groups to display moderate anxiety during the interview compared with the NC children, Χ^2^ (4) = 5.55, *p* = 0.06.

Inter-rater reliability for flat affect ratings, as calculated by kappa, was 0.68. In this offspring sample, flat affect was less frequently observed than anxiety, inappropriate affect, or emotional withdrawal. The three offspring groups did not differ significantly in terms of the proportion of children displaying flat affect, Χ^2^ (2) = 1.14, *p* = 0.57 and there was no gender difference in this regard.

The kappa value for inter-rater reliability for inappropriate affect was 0.85. HRSz and HRAff children were both more likely to display inappropriate affect than NC children, Χ^2^ (2) = 21.35, *p* < 0.001, and Cramer’s *V* = 0.35. The HRSz group did not show gender differences in terms of inappropriate affect, Χ^2^ (1) = 0.05, n.s. Similarly, there were no gender differences in the HRAff, or NC, group, Fisher’s Exact Test, *p* = 0.14 and 0.25, respectively.

Inter-rater reliability for emotional withdrawal ratings, as measured by Cohen’s kappa, was 0.79. A significantly larger proportion of the HRSz children displayed emotional withdrawal than HRAff and NC children, Χ^2^ (2) = 16.19, *p* < 0.001, and Cramer’s *V* = 0.31. Males in the NC offspring group were more likely to be rated as showing emotional withdrawal than the females, Χ^2^ (1) = 4.12, *p* < 0.05. This was not the case with either the HRSz group [Χ^2^ (1) = 1.91, n.s.], or the HRAff group [Fisher’s exact test, *p* = 0.48].

### 3.3 Group comparisons by adulthood diagnostic Outcome

Table 4 presents the distribution of participants by offspring group and adulthood outcome diagnoses. Of the twenty offspring who developed either schizophrenia-related psychoses or affective psychoses in adulthood, 65% (13) were from the HRSz group and 30% (*n* = 6) were from the HRAff group.

Given the relatively small size of some of the outcome diagnostic groups, we compared the groups in terms of psychotic disorders versus nonpsychotic disorders, and psychiatric disorder versus no psychiatric disorder. The mean ratings of emotion expressivity by adult diagnostic outcome and interview question appear in Table 5. None of the displays of discrete emotions during mid-childhood were associated with adult diagnostic outcomes.

**Fig. 2:**
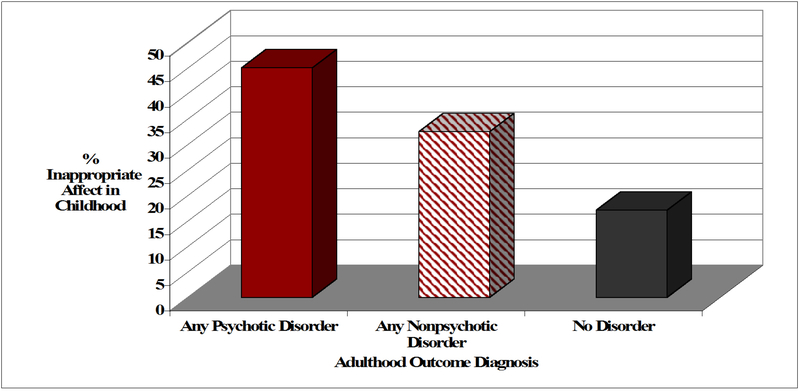
The percentage of participants who displayed inappropriate affect during the MHAF when interviewed during mid-childhood who later had a psychiatric outcome as determined by a structured clinical interview in adulthood.

We observed a significant association between a display of inappropriate affect during mid-childhood and adulthood psychiatric outcome. As indicatedin Fig. 2, children having a later psychiatric outcome were significantly more likely to exhibit inappropriate affect during the interview, Χ^2^ (2) = 8.20, *p* = 0.02, and Cramer’s *V* = 0.20. Children showing flat affect during the mid-childhood MHAF were not more likely to develop a psychiatric disorder in adulthood, Χ^2^ (2) = 0.01, *p* = 0.99. The association between ratings of childhood emotional withdrawal and adulthood psychiatric outcome did not reach significance, Χ^2^ (2) = 3.92, n.s. Children manifesting moderate anxiety during the interviews appeared more likely to receive an adulthood psychiatric diagnosis than children manifesting either no,or mild, anxiety during the interviews, Χ^2^ (2) = 5.38, *p* = 0.07. The difference was not statistically significant.

## 4 DISCUSSION

### 4.1 Advantages and significance of present study

This is the first study to investigate the emotional expressivity of at-risk individuals from childhood through the period of greatest risk for schizophrenia that also includes a non-psychotic psychiatric comparison group. One of the advantages of this study is the focus on a fine-grained analysis of discrete emotions as well as inclusion of molar ratings of emotional engagement. Our ratings of affective and social behaviors in the context of a social encounter provide further indications that individuals at genetic risk for schizophrenia display social and affective deficits early in their development.

We have identified differences in a child’s emotional expressivity during a semi-structured interview that are associated with their genetic risk for psychiatric disorder. Specifically, the HRSz group showed more negative affect and less positive affect in response to questions about their worst experience and their self-concept, respectively. This suggests that HRSz children may genuinely experience more negative affect than children of healthy controls or children of parents with affective disorders.

Studies ^[50–53]^ have indicated that social anhedonia is a significant risk factor for schizophrenia-spectrum disorders. The finding that HRSz individuals display less positive affect in response to questions about their self-concept may be relevant to the developmental ontogeny of social anhedonia in schizotypal individuals. The HRSz offspring group showed significantly less positive affect when questioned about their self-concept, *i.e.*, how much did they like themselves or what would they change about themselves. If individuals at risk for schizophrenia experience an altered hedonic response to their own self-concept, this may negatively impact the development of hedonic associations related to social stimuli. More research is needed to clarify the potential role of a reduced hedonic representation of one’s self-concept in the development of social anhedonia.

These findings of impaired affective functioning in HRSz children are consistent with earlier reports based upon the genetic high-risk method ^[7]^ as well as the archival-observational method ^[5,6]^ indicating greater negative emotion being associated with preschizophrenic children. In contrast to Walker and colleagues ^[5,6]^, we did not find that adulthood schizophrenia outcome could be predicted on the basis of facial expressions of comparatively less positive affect, or greater negative affect, during childhood. While we observed significant differences in the frequency of affective displays in response to affect-eliciting questions, these differences did not predict later psychiatric outcomes. We attribute the differences in the findings in part to a different methodology (*i.e.*, the archival-observational method using forced-choice versus prospective longitudinal method using a coding system that provides for assessment of several emotions simultaneously).

More of the high-risk children displayed inappropriate affect than the normal control children. It is noteworthy that in the present study we used a measure of inappropriate affect that combined both positive and negative affect. In future investigations, it would be useful to differentiate the types of inappropriate affect displayed by the child. It is possible that the HRSz and the HRAff groups exhibited inappropriate affect in different ways and for different reasons. For example, one might speculate that the HRAff children may display inappropriate positive affect as a learned response in an attempt to comfort their parents ^[54]^. In contrast, HRSz children may display inappropriate negative affect as a manifestation of neural dysregulation ^[55]^.

One of the advantages of the prospective high-risk design is that it permits the study of behavioral antecedents of adulthood psychiatric outcomes. Our observation of an association between inappropriate affect during childhood and later adulthood diagnostic outcome implies a role for inappropriate affect as a psychopathology risk-related factor. Individuals who developed a psychiatric disorder in adulthood, whether psychotic or nonpsychotic, showed inappropriate affect more frequently than children who did not develop psychiatric illness. This suggests that inappropriate affect in the offspring of psychiatrically ill parents may be a nonspecific indication of risk for the development of adulthood psychopathology.

Furthermore, we observed that HRSz children were more likely to exhibit emotional withdrawal than the children in the other two groups. This suggests that emotional withdrawal may be specific indicator of risk for the later development of schizophrenia. The New York High Risk Project is the first study in which HRSz children have been independently observed to display significantly more emotional withdrawal compared to other groups of at-risk children. Our finding of increased emotional withdrawal being associated with genetic risk for schizophrenia shows considerable consistency with the results of prior investigations of emotional problems as antecedents to schizophrenia ^[16,31,56,57]^.

Our findings are consistent with earlier indications that individuals at genetic risk for schizophrenia manifest observable signs of schizotypy, such as emotional withdrawal ^[58,59]^ and inappropriate affect ^[58]^ at relatively early ages. Our findings extend the literature by suggesting that some of these signs may be specifically related to a schizophrenia diathesis. Despite differences in methodology, the NYHRP data are consistent with findings based upon retrospective parental reports ^[33]^ which indicate that emotional withdrawal in mid-childhood is associated with heightened risk for a schizophrenia-related outcomes in adulthood. Our findings regarding emotional withdrawal are also in line with data from prodromal studies ^[35,36]^ in which individuals with non-affective psychosis typically display social withdrawal in the years preceding their first episode of psychosis.

### 4.2 Limitations, strengths, and future directions

One limitation of the present study is that it was unable to explore gender differences within the at-risk groups. The relationship between gender and genetic risk in terms of emotional expressivity deserves further study, given the related findings of Walker and colleagues ^[5,6]^ as well as observations that children display gender-linked roles early in development, with gender differences in socio-emotional development appearing in childhood ^[60]^. Nonwhite children were not included in the original samples in the NYHRP, which started in the early 1970s. Conclusions are thus limited to Caucasian populations.

Sample sizes varied according to the question. Due to the quality of the recordings as well as some variations in the semi-structured interviews, not all children were asked all the same questions. This investigation is a secondary analysis of a well-known data set. As expected, when data are used to explore questions that were not salient at the time of the study design, we could not expect that all the relevant data would be available.

We did not measure parent-child attachment, so it is possible that increased negative affect and emotional withdrawal could be related to poorer attachment, rather than simply a genetic endophenotype. We did not assess the level of trauma experienced by the children. Prior work suggests that traumatic experiences during childhood may contribute to the development and/ or maintenance of psychosis ^[34]^. Future research in this area would benefit from measuring level and severity of childhood traumatic events as well as quality of parent-child attachment. Finally, we cannot rule out environmental factor effects that may have contributed to group differences in affective expressivity that we observed. Given the presence of serious psychopathology in the families of the atrisk offspring, it would not have been unreasonable to see increased displays of negative affect overall. However, that was not the case. Rather, we observed that a disproportionate number of children in both high-risk groups exhibited inappropriate affect, relative to the children in the NC group.

## 5 CONCLUSION

The present study demonstrated that childhood emotional expressivity is associated with later psychiatric outcomes. These data are significant for two reasons: first, they provide convergent evidence for affective deficits being a key feature of schizophrenia-spectrum disorders; and second, they suggest that the genetic diathesis associated with schizophrenia may become manifest early in the life span, years prior to the onset of an overt psychiatric disorder.

## Figures and Tables

**Table 1.** Demographic characteristics of the three offspring groups.

Characteristic	All	HRSz	HRAff	NC

N	173	61	54	58
Gender				
Male	89	33	17	39
Female	84	28	37	19
Age^a^	10.65 ± 1.8	10.69 ± 1.9	10.74 ± 1.7	10.53 ± 1.9

a Mean ± SD.

**Table 2.** Mean ratings of affective expressivity by offspring group^a^.

Interview question^b^	HRSz	HRAff	NC	*p*

Best/Happiest^c^	5.22 ± 1.3	5.67 ± 0.8	5.59 ± 0.75	n.s.
Worst/Saddest^c^	3.50 ± 1.46	4.23 ± 1.83	4.38 ± 1.36	*
Change in Self^c^	4.67 ± 1.47	5.58 ± 0.84	5.45 ± 1.12	*
General Mood^c^	4.85 ± 1.00	5.24 ± 1.23	5.11 ± 1.16	n.s.
DayDreaming^c^	4.96 ± 1.54	5.20 ± 1.15	5.27 ± 0.77	n.s.
Dreams at Night^c^	5.31 ± 1.05	5.48 ± 1.37	5.15 ± 1.17	n.s

a The ratings are on a 7-point scale. 1 denotes the most strongly negative emotional expression and 7 denotes the most strongly positive emotional expression.

b A child’s emotional expressions were rated during their response to semi-structured interview probes by child psychiatrists. See the text for questions. HRSz = offspring at risk for schizophrenia; HRAff = offspring at risk for affective disorders; and NC = offspring of psychiatrically-healthy parents.

c The n’s varied slightly in terms of the availability of useable data for analyses of discrete emotions. The n’s ranged from 25–48 for the HRSz group, from 19–44 for the HRAff group, and from 22–47 for the NC group.

* p < 0.05.

**Table 3.** Number (and percent) displaying affective behavior by offspring group.

Behavior^a, b^	HRSz	HRAff	NC

Anxiety			
No	10 (16.4)	8 (14.8)	13 (22.4)
Mild	24 (39.3)	25 (46.3)	31 (53.4)
Moderate	27 (44.3)	21 (38.9)	14 (24.1)
Any anxiety	51 (83.6)	46 (85.2)	45 (77.6)
Flat Affect	11 (18.0)	6 (11.1)	8 (13.8)
Inappropriate Affect	23 (37.7)	21 (38.9)	3 (15.8)
Emotional Withdrawal	32 (52.5)	10 (18.5)	16 (27.6)

a Operational definitions for these behaviors appear in Methods.

b Cohen’s kappa values for interrater agreement appear in Results. HRSz = offspring at risk for schizophrenia; HRAff = offspring at risk for affective disorder; and, NC = offspring of psychiatrically healthy parents.

**Table 4.** Frequency of participants by offspring group and adulthood diagnostic outcome.

Adult diagnostic outcome	Offspring group			
	HRSz	HRAff	NC	Total

Any psychotic diagnosis^a^	13	6	1	20
Any nonpsychotic Axis I diagnosis	27	35	15	77
No diagnosis (Healthy)	21	13	42	76
Total	61	54	58	173

HRSz = high-risk for schizophrenia; HRAff = high-risk for affective disorders; NC = normal controls.

a Any psychotic diagnosis included schizophrenia-related psychotic disorders and affective psychoses; Nonpsychotic Axis I diagnosis included nonpsychotic major affective disorders, anxiety disorders, and substance abuse disorders.

**Table 5.** Emotion expressivity mean ratings by adult diagnostic outcome group.

Interview question	Any psychotic disorder	Any nonpsychotic disorder	No psychiatric disorder	*p*

Best/Happiest	5.42 ± 1.0	5.57 ± 0.8	5.40 ± 1.2	n.s.
Worst/Saddest^c^	3.93 ± 1.75	3.84 ± 1.55	4.25 ± 1.60	n.s.
Change in Self	5.14 ± 1.22	5.33 ± 1.18	4.97 ± 1.40	n.s.
General Mood^c^	4.80 ± 1.10	4.98 ± 1.28	5.16 ± 0.98	n.s.
Daydreaming	5.71 ± 0.95	5.09 ± 1.20	5.04 ± 1.27	n.s.
Dreams at night^c^	5.82 ± 0.87	5.28 ± 1.37	5.22 ± 1.03	n.s.

The ratings are on a 7 point scale, where 1 denotes the most strongly negative emotional expression and 7 denotes the most strongly positive emotional expression. The child’s emotional expressions were rated during their response to semi-structured interview probes by child psychiatrists. See text for questions. The n’s varied slightly in terms of the availability of useable data for analyses of discrete emotions. n’s ranged from 93 for the question regarding a child’s general mood to 139 for the query regarding the child’s worst, or saddest, experience.

* *p* < 0.05.

## ABBREVIATIONS

NYHRP: New York High-Risk Project
HRSz: high risk for Schizophrenia
HRAff: high risk for Affective disorders
NC: normal comparison
MHAF: Mental Health Assessment Form
SADS-L: Schedule for Affective Disorders and Schizophrenia, Lifetime version
PDE: Personality Disorders Examination
ANOVA: Analysis of Variance
ICC: Intra-class correlation
